# Abnormal Dexamethasone Suppression Tests in a Rifapentine-Treated Patient With Primary Aldosteronism

**DOI:** 10.3389/fendo.2020.00593

**Published:** 2020-09-04

**Authors:** Hongman Wang, Ying Song, Zhixin Xu, Ying Jing, Wenwen He, Zhengping Feng, Qifu Li, Shumin Yang

**Affiliations:** ^1^Department of Endocrinology, Chongqing General Hospital, Chongqing, China; ^2^Department of Endocrinology, The First Affiliated Hospital of Chongqing Medical University, Chongqing, China

**Keywords:** dexamethasone suppression tests, primary aldosteronism, rifapentine, hypercortisolism, hypertension

## Abstract

Aldosterone-producing adenoma (APA) is a main cause of primary aldosteronism (PA). Given that a large benign-appearing unilateral masse (>1 cm in diameter) may represent an aldosterone and cortisol-co-secreting adenoma, dexamethasone suppression testing is required in such patients to exclude or confirm the diagnosis of hypercortisolism. Tuberculosis is highly prevalent in China, and rifamycins are often used in these patients. Rifapentine belongs to the rifamycin family, and we herein for the first time report a case of misdiagnosis of hypercortisolism due to rifapentine use in a patient with APA. Thus, in patients treated with rifapentine, diagnosis of hypercortisolism based on dexamethasone suppression tests can be very misleading.

## Introduction

Primary aldosteronism (PA) is a common cause of secondary hypertension. Aldosterone-producing adenoma (APA) is a main cause of PA, accounting for ~1/3 of PA (1). Coexistence with subclinical Cushing's syndrome is not uncommon in PA (2–4). In addition, a large benign-appearing unilateral masse (>1 cm in diameter) may represent an aldosterone- and cortisol-co-secreting adenoma, so dexamethasone suppression testing (DST) is mandatorily required in these patients to exclude or confirm Cushing's syndrome (5). Tuberculosis is highly prevalent in China, and rifamycins are often used in these patients (6). Previous studies reported that rifampicin could cause misleading results of DST (7). Rifapentine belongs to the rifamycin family, and we herein for the first time report a case of misdiagnosis of hypercortisolism due to rifapentine use in a patient with PA.

## Case Presentation

The patient was a 50-year-old Chinese man who was diagnosed with hypertension at the age of 42 years. The patient was treated with felodipine alone after he was diagnosed with hypertension, and his blood pressure was ~140–150/90–100 mmHg with felodipine treatment. Approximately 1 month earlier, he felt dizzy and his self-measured blood pressure was found to be 190/120 mmHg. He went to the hospital and was prescribed nifedipine 30 mg three times a day, irbesartan hydrochlorothiazide (150 mg/12.5 mg) one tablet twice a day and, metoprolol 47.5 mg/day. He occasionally measured his blood pressure, which was ~130–150/80–90 mmHg.

The patient had type 2 diabetes mellitus for 6 years, and he currently took gliclazide 30 mg/d and metformin 500 mg twice a day. He was diagnosed with tuberculosis 2 months earlier. Since then, he was regularly taking ethambutol 750 mg/day, isoniazid 100 mg/day, and rifapentine 600 mg twice a day.

**Figure 1:**
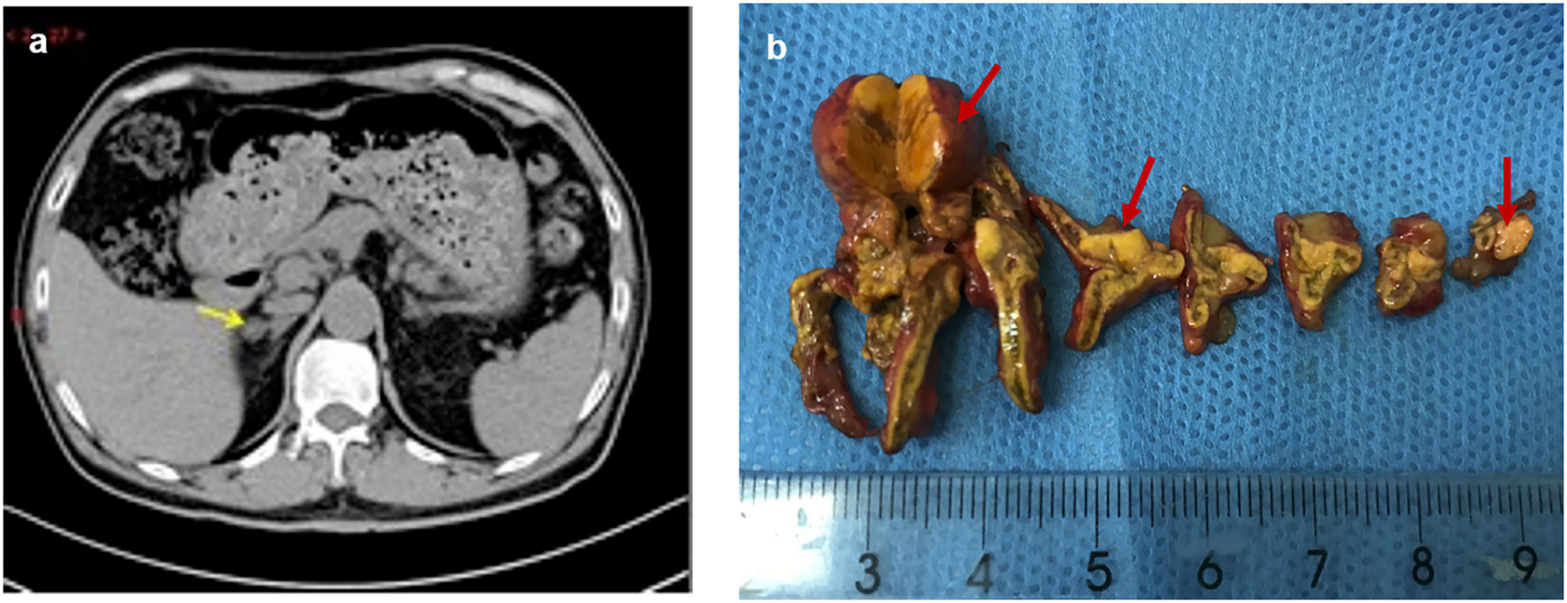
Preoperative adrenal CT and post-operative specimen of the patient. The right adrenal nodule with low density (arrow) was identified on CT **(a)**, and surgical resection specimen revealed three nodules (red arrow) **(b)**.

Due to his severe hypertension, he was referred to the endocrinology department. The laboratory test showed hypokalemia of 3.1 mmol/L (reference: 3.5–5.5 mmol/L). Two weeks after changing his antihypertensive therapy to nifedipine-controlled released tablet 30 mg/day and doxazosin 4 mg/day, laboratory tests showed a high plasma aldosterone concentration (PAC) of 17.4 ng/dl (to convert to pmol/L, multiply by 27.74), a low plasma renin concentration (PRC) of 4.2 mIU/l (to convert to pmol/L, multiply by 0.0375), and a high aldosterone/renin ratio (ARR) of 41 (ng/dl)/(mIU/l) (reference <2.0) (Table 1), which suggested PA. The saline infusion test and the captopril challenge test (Table 1) confirmed the diagnosis of PA. Adrenal computed tomography (CT) was performed, and a right adrenal nodule (13 mm in diameter) with low density was identified (Figure 1).

**Table 1:** Laboratory test results of the patient before surgery.

Plasma potassium (mmol/l)	3.1
24 h urine potassium (mmol)	60.3
PAC (ng/dL)	17.4
PRC (mIU/L)	4.2
PAC post-CCT (ng/dL)	109
PAC post-SIT (ng/dL)	113
Plasma cortisol at 8 a.m. (nmol/L)	596.9
Plasma cortisol at 4 p.m. (nmol/L)	158.2
Plasma cortisol at 0 a.m. (nmol/L)	89.8
Plasma ACTH at 8 a.m. (pg/mL)	23.4
Plasma ACTH at 4 p.m. (pg/mL)	5.0
Plasma ACTH at 0 a.m. (pg/mL)	4.8
24 h urine-free cortisol (nmol)	774.7
Plasma cortisol after 1 mg overnight DST (nmol/L)	218.7
Plasma cortisol after 48-h low-dose DST (nmol/L)	384.5

*PAC, plasma aldosterone concentration; PRC, plasma renin concentration; ACTH, adrenocorticotropic hormone; CCT, captopril challenge test; SIT, saline infusion test; DST, dexamethasone suppression test*.

**Figure 2:**
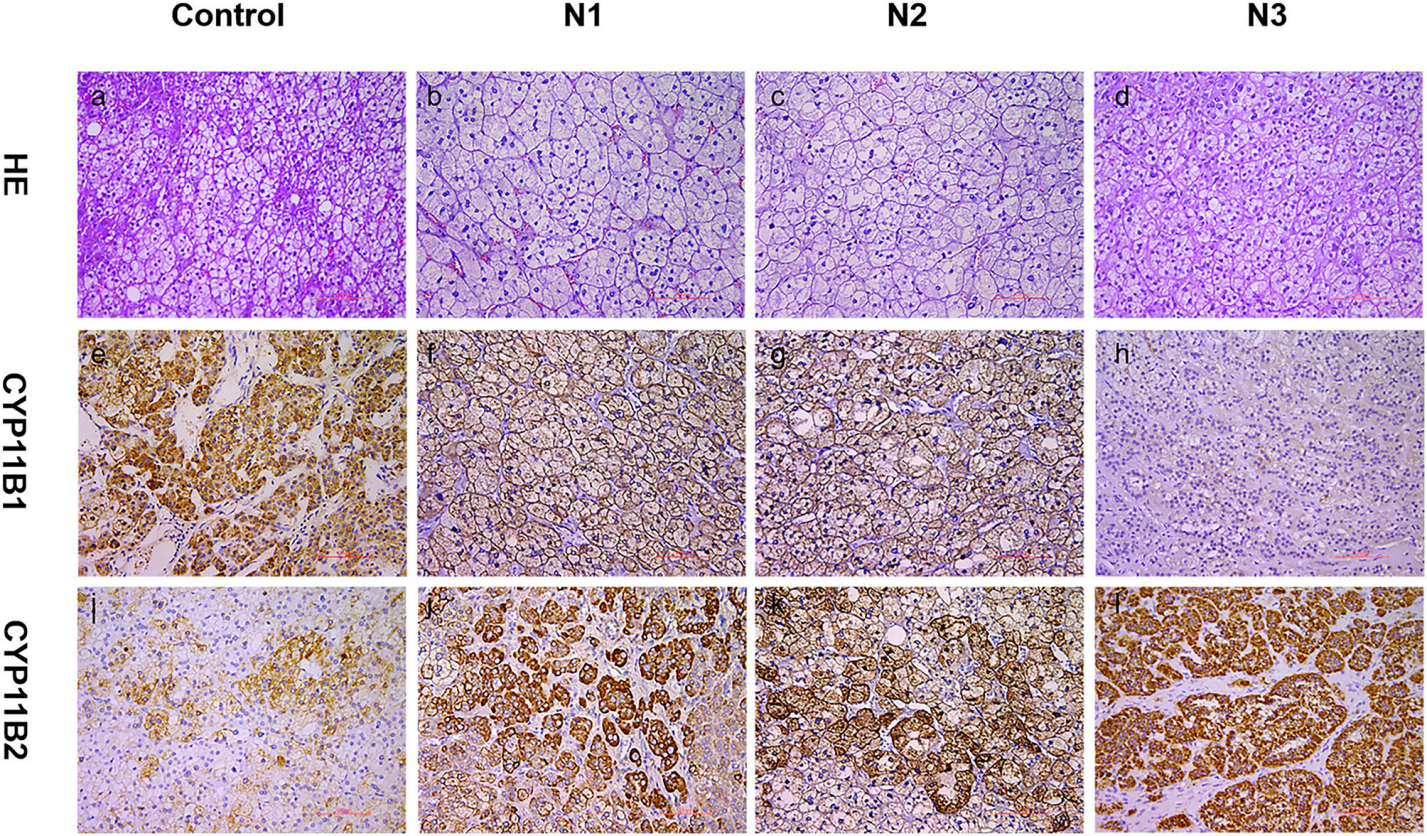
Expression of CYP11B1/2 in three nodules of patients. Histological and immunohistochemical staining of three nodules (N1, nodule 1; N2, nodule 2; N3, nodule 3) of this patient and cortisol-producing adenoma from another patient (control, as a reference for positive CYP11B2 staining). HE, hematoxylin–eosin staining **(a–d)**; CYP11B2: immunohistochemical staining of aldosterone synthase **(i–l)**; CYP11B1: immunohistochemical staining of 11β-hydroxylase **(e–h)**; 200× magnification.

**Figure 3:**
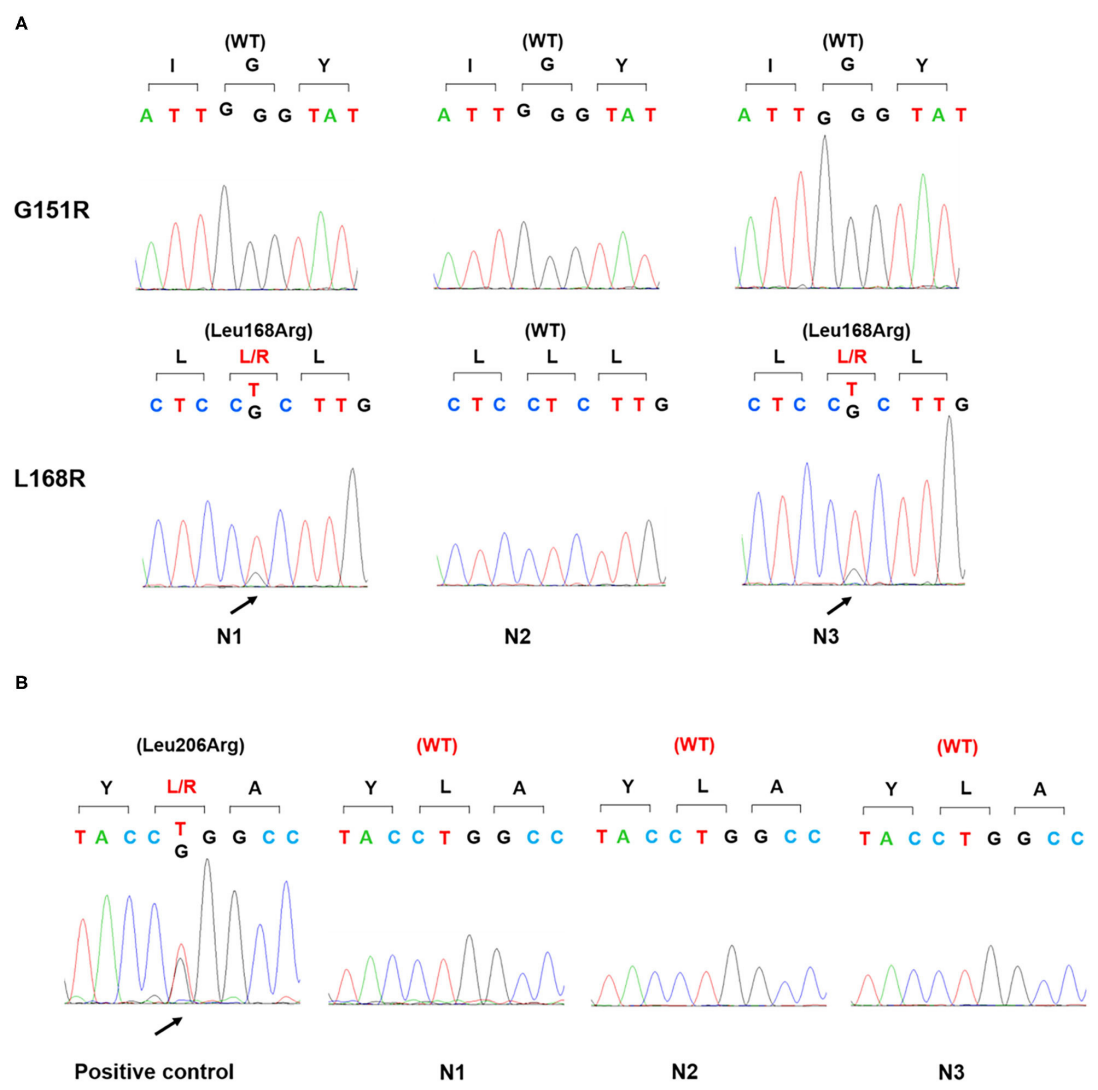
Gene sequence of KCNJ5 and PRKACA in three nodules. Genomic DNA was isolated from the patient's three adrenal nodules and amplified by PCR. Sanger sequencing detected a p.Leu168Arg mutation of KCNJ5 gene **(A)** in two nodules (N1, N3) and no p.Leu206Arg mutation of PRKACA gene in all three nodules. Positive control: a cortisol-producing adenoma harboring a p.Leu206Arg mutation of PRKACA gene **(B)**; WT, wild type.

Physical examination revealed no full moon face, no centripetal obesity, and no skin purple skin striae. His 24-h urine-free cortisol, plasma cortisol, and plasma adrenocorticotropic hormone levels were normal (Table 1). However, 1 mg overnight and 2 mg/d for 48 h DST yielded positive results (>138 nmol/l). Plasma cortisol levels at 8 a.m. after 1 and 2 mg dexamethasone administration were 218.7 and 384.5 nmol/l, respectively (Table 1). Because some patients with Cushing's syndrome have inconsistent 24-h urine-free cortisol and DST results, subclinical Cushing's syndrome was considered in this patient, although his 24-h urine-free cortisol was not high. According to the guideline, patients younger than 35 years, with spontaneous hypokalemia, marked excess aldosterone, and unilateral adrenal lesions with radiological features consistent with a cortical adenoma on adrenal CT scan may not need adrenal vein sampling before proceeding to unilateral adrenalectomy (5). For this patient, given PAC and PRC levels as well as his age, adrenal venous sampling was strongly recommended but the patient refused. Laparoscopic total right adrenalectomy was performed. Three nodules were found in the right adrenal resection specimen, and only the largest nodule was observed by CT (Figure 1). Histology showed clear cells with vacuolated cytoplasm. Immunohistochemical staining for the enzymes involved in aldosterone (CYP11B2) and cortisol (CYP11B1) (Figure 2) biosynthesis suggested that all the three nodules secreted aldosterone, whereas none of them secreted cortisol. KCNJ5 mutations were found in two of the three nodules, whereas no PRKACA mutations were found (Figure 3).

One month after surgery, the patient's serum potassium was normal. The PAC decreased to 3.9 ng/dl and PRC increased to 9.2 mIU/L, resulting in a normal ARR, 0.4 (ng/dl)/(mIU/l) (Table 2). However, the patient was still found with resistant hypertension. Despite administration of nifedipine-controlled released tablet 30 mg twice a day, irbesartan hydrochlorothiazide (150 mg/12.5 mg) one tablet twice a day, and metoprolol 47.5 mg/day, his blood pressure was remained uncontrolled. In addition, 1 mg overnight DST yielded positive results (with 8 a.m. plasma cortisol levels of 369.8 nmol/l after administration of 1 mg dexamethasone). This situation disturbed the patients, and we discussed the possible cause with him. Rifapentine-induced false positive in DST as well as uncontrolled blood pressure was considered. Two months after he completed the tuberculosis treatment, 1 mg overnight DST results were negative (8 a.m. plasma cortisol levels of 29.8 nmol/l after administration of 1 mg dexamethasone). His blood pressure was well-controlled with nifedipine alone (30 mg/day). Finally, the patient was diagnosed with right APA and, he was satisfied with the treatment effect.

**Table 2:** Follow-up results of the patient after surgery.

	**1 month**	**6 months**
Use of rifapentine	Yes	No (discontinued for 2 months)
Anti-hypertension medication	Nifedipine 30 mg twice a day, irbesartan hydrochlorothiazide (150 mg/12.5 mg) twice a day and metoprolol 47.5 mg/day	Nifedipine 30 mg/day
Blood pressure (mmHg)	150–159/99–103	130–135/80–90
Plasma potassium (mmol/l)	4.3	4.0
PAC (ng/dL)	3.9	5.4
PRC (mIU/L)	9.2	13.3
Plasma cortisol at 8 a.m. (nmol/L)	446.8	–
Plasma ACTH at 8 a.m. (pg/mL)	15.1	–
Plasma cortisol after 1 mg overnight DST (nmol/L)	369.8	29.8

*PAC, plasma aldosterone concentration; PRC, plasma renin concentration; ACTH, adrenocorticotropic hormone; DST, dexamethasone suppression test*.

## Patient Consent

Informed consent was obtained from the patient for publication of the case report and accompanying images.

## Discussion

This patient was admitted for PA screening given his severe hypertension, and he was confirmed with hyperaldosteronism. Although he had no clinical signs of Cushing's syndrome, he was subsequently subjected to DST due to the mass on adrenal CT while being treated with anti-tuberculosis drugs including rifapentine. He was unaware that rifapentine could profoundly affect the interpretation of test results. As he lacked the typical clinical features of Cushing's syndrome, we considered a diagnosis of subclinical Cushing's syndrome based on the results from DST. Unexpectedly, DST was still abnormal after surgery. Besides, immunohistochemical staining for CYP11B1 and PRKACA analysis did not support the diagnosis of Cushing's syndrome. At that time, we began to realize the potential for a misdiagnosis of Cushing's syndrome. In addition, his intact diurnal variation of cortisol secretion, unsuppressed ACTH, and normal 24-h urine-free cortisol also did not support the diagnosis of Cushing's syndrome.

Rifamycins are widely used drugs for the treatment of tuberculosis which is highly prevalent in China (6). Rifampicin is a potent inducer of the hepatic mixed oxygenase enzymes involved in drug metabolism. Patients receiving rifampicin have increased liver cytochrome P450 activity, which might accelerate the metabolism of other drugs (8). Rifampicin-induced adrenal crisis in patients with adrenal failure receiving corticosteroid replacement has been reported in several studies (9–11). A Japanese report found that in patients receiving rifampicin therapy for tuberculosis, the half-life of dexamethasone decreased three-fold and the clearance rate increased five-fold (12). In addition, Kyriazopoulou et al. found that plasma dexamethasone concentrations were much lower (range, 1.2–4.8 nmol/L) than the normal range (11.2–23.4 nmol/L) after rifampicin treatment (13). In a study of normal subjects, dexamethasone administration before rifampicin treatment completely suppressed serum cortisol in all subjects (32 ± 21 nmol/L), but dexamethasone was ineffective after rifampicin treatment for 10 days (434 ± 82 nmol/L) (13). Abdullah et al. reported abnormal results of 1 mg overnight DST and 2 mg/day for 48 h in a patient with subtle signs of Cushing's syndrome who was also being treated with rifampicin for tuberculosis (7). When the patient proceeded to the 48-h high-dose DST, serum cortisol levels were suppressed to 31 nmol/L.

There are no reports on abnormal DST in subjects treated with rifapentine. Given that rifapentine is also an inducer of cytochrome P450, we hypothesize that the removal of dexamethasone by the liver was accelerated with rifapentine treatment, and dexamethasone failed to suppress serum cortisol in our patient. The DST was abnormal. If this finding was applied in this patient, it could mislead the physician into diagnosing non-existent Cushing's syndrome. Although no study proves the time required to restore normal response to DST after discontinuing rifapentine therapy, it would be prudent to stop rifapentine therapy for 14 days at least before performing DST. This judgment is based on the drug instruction that the enzyme activity will be restored to normal levels 14 days after discontinuing rifapentine (14, 15). Currently, rifabutin is frequently used as it has a much longer half-life than rifampin and rifapentine. Therefore, the time required to restore a normal response to DST after discontinuing rifabutin therapy should be much longer. In addition to rifamycins, other drugs that could influence the DST results should also be considered. Dexamethasone is a substrate for CYP3A4, and inhibitors of CYP3A4 (e.g., diltiazem, verapamil, amiodarone, clarithromycin, ciprofloxacin, fluconazole, ketoconazole, itraconazole, cimetidine, nelfinavir, imatinib, and mifepristone) dexamethasone blood concentrations. In contrast, inducers of CYP3A457 (e.g., carbamazepine, sodium phenytoin, and pioglitazone) could decrease dexamethasone blood concentration during DST, resulting in false-negative or false-positive results (https://drug-interactions.medicine.iu.edu/MainTable.aspx).

Another interesting finding in this case is that rifapentine seems to have influenced the blood pressure of our patient. From his medical history, we knew that before treatment with rifapentine his blood pressure was 140–150/80–90 mmHg with felodipine 5 mg per day. Approximately 1 month after initiation of rifapentine, he began to feel dizzy. Then, he was subsequently diagnosed with severe hypertension, which caused him to present to the hospital. Studies concerned with the effect of rifapentine or rifampicin on blood pressure are limited. Simkins et al. reported that 22% of renal transplant candidates with baseline blood pressure <140/90 mmHg developed severe hypertension after starting rifapentine and that most of these events occurred during the first 6 weeks of treatment (16). It is possible that rifapentine can decrease the efficacy of certain antihypertensive drugs through the induction of cytochrome P450 (17). In fact, even after resection of the APA, our patient still exhibited severe hypertension. However, when he discontinued rifapentine, his blood pressure was well-controlled with nifedipine alone, suggesting a possible influence of rifapentine on blood pressure.

To the best of our knowledge, this is the first reported case of misdiagnosis with hypercortisolism due to rifapentine in a patient with aldosterone-producing adenomas. This case might provide some useful information for clinicians. However, the lack of dexamethasone concentrations assay is a limitation of our study.

## Conclusion

When screening patients for Cushing's syndrome, it is important to be aware of potential drug–drug interaction with dexamethasone, which may lead to false-positive results and erratic diagnoses. In patients using rifamycins, such as rifampicin and rifapentine, diagnosis of Cushing's syndrome should be prudent. It would be advisable to stop rifapentine therapy for at least 2 weeks before performing DST.

## Data Availability Statement

All datasets generated for this study are included in the article/supplementary material.

## Ethics Statement

The studies involving human participants were reviewed and approved by the First Affiliated Hospital of Chongqing Medical University. The patients/participants provided their written informed consent to participate in this study. Written informed consent was obtained from the individual(s) for the publication of any potentially identifiable images or data included in this article.

## Author Contributions

SY, QL, and HW designed the study, oversaw the data collection, and wrote the manuscript. YS, ZX, YJ, WH, and ZF contributed to the writing of the manuscript. SY and QL were the guarantor of this work, had full access to all the data in the study, and takes responsibility for the integrity of the data. All authors contributed to the article and approved the submitted version.

## Conflict of Interest

The authors declare that the research was conducted in the absence of any commercial or financial relationships that could be construed as a potential conflict of interest.

## References

[1] Monticone S Burrello J Tizzani D Bertello C Viola A Buffolo F. Prevalence and clinical manifestations of primary aldosteronism encountered in primary care practice. J Am Coll Cardiol. (2017) 69:1811–20. 10.1016/j.jacc.2017.01.052 28385310

[2] Hiraishi K Yoshimoto T Tsuchiya K Minami I Doi M Izumiyama H. Clinicopathological features of primary aldosteronism associated with subclinical Cushing's syndrome. Endocr J. (2011) 58:543–51. 10.1507/endocrj.K10E-402 21521926

[3] Fallo F Castellano I Gomez-Sanchez CE Rhayem Y Pilon C Vicennati V. Histopathological and genetic characterization of aldosterone-producing adenomas with concurrent subclinical cortisol hypersecretion: a case series. Endocrine. (2017) 58:503–12. 10.1007/s12020-017-1295-4 28405879 PMC5638684

[4] Lau JH Sze WC Reznek RH Matson M Sahdev A Carpenter R. A prospective evaluation of postural stimulation testing, computed tomography and adrenal vein sampling in the differential diagnosis of primary aldosteronism. Clin Endocrinol. (2012) 76:182–8. 10.1111/j.1365-2265.2011.04202.x 21895732

[5] Funder JW Carey RM Mantero F Murad MH Reincke M Shibata H. The management of primary aldosteronism: case detection, diagnosis, and treatment: an endocrine society clinical practice guideline. J Clin Endocrinol Metab. (2016) 101:1889–916. 10.1210/jc.2015-4061 26934393

[6] Yan L Kan X Zhu L Xu K Yin J Jie L. Short-course regimen for subsequent treatment of pulmonary tuberculosis: a prospective, randomized, controlled multicenter clinical trial in China. Clin Ther. (2018) 40:440–9. 10.1016/j.clinthera.2018.01.013 29519716

[7] Abdullah HN Nowalid WK. Abnormal dexamethasone suppression tests in a rifampicin-treated patient with suspected Cushing's syndrome. Endokrynol Pol. (2010) 61:706–9. 21104646

[8] Miguet JP Mavier P Soussy CJ Dhumeaux D. Induction of hepatic microsomal enzymes after brief administration of rifampicin in man. Gastroenterology. (1977) 72:924–6. 10.1016/S0016-5085(77)80212-6 849823

[9] Kyriazopoulou V Parparousi O Vagenakis AG. Rifampicin-induced adrenal crisis in addisonian patients receiving corticosteroid replacement therapy. J Clin Endocrinol Metab. (1984) 59:1204–6. 10.1210/jcem-59-6-1204 6490796

[10] Elansary EH Earis JE. Rifampicin and adrenal crisis. Br Med J. (1983) 286:1861–2. 10.1136/bmj.286.6381.1861-a 6407604 PMC1547783

[11] Ediger SK Isley WL. Rifampicin-induced adrenal insufficiency in the acquired immunodeficiency syndrome: difficulties in diagnosis and treatment. Postgrad Med J. (1988) 64:405–6. 10.1136/pgmj.64.751.405 3200786 PMC2428690

[12] Kawai S. A comparative study of the accelerated metabolism of cortisol, prednisolone and dexamethasone in patients under rifampicin therapy. Nihon Naibunpi Gakkai Zasshi. (1985) 61:145–61. 10.1507/endocrine1927.61.3_145 4018307

[13] Kyriazopoulou V Vagenakis AG. Abnormal overnight dexamethasone suppression test in subjects receiving rifampicin therapy. J Clin Endocrinol Metab. (1992) 75:315–7. 10.1210/jcem.75.1.1619024 1619024

[14] Baciewicz AM Chrisman CR Finch CK Self TH. Update on rifampin, rifabutin, and rifapentine drug interactions. Curr Med Res Opin. (2013) 29:1–12. 10.1185/03007995.2012.747952 23136913

[15] Irony I Kater CE Biglieri EG Shackleton CH. Correctable subsets of primary aldosteronism. Primary adrenal hyperplasia and renin responsive adenoma. Am J Hypertens. (1990) 3:576–82. 10.1093/ajh/3.7.576 2194512

[16] Simkins J Morris MI Abbo LM Camargo JF. Severe hypertension after initiation of rifapentine/isoniazid for latent tuberculosis in renal transplant candidates. Transpl Int. (2017) 30:108–9. 10.1111/tri.12881 28032405

[17] Cordeanu EM Gaertner S Faller A Mirea C Lessinger JM Kemmel V. Rifampicin reverses nicardipine effect inducing uncontrolled essential hypertension. Fundam Clin Pharmacol. (2017) 31:587–9. 10.1111/fcp.12292 28407303

